# ASCO Leadership Development Program: International Perspectives

**DOI:** 10.1200/JGO.18.00014

**Published:** 2018-04-13

**Authors:** Roselle De Guzman, Monica Malik, Gilberto de Lima Lopes, Rebecca Alexandra Dent, Shaheenah Dawood

**Affiliations:** **Roselle De Guzman**, St Lukes Medical Center, Quezon City, Philippines; **Monica Malik**, Nizam’s Institute of Medical Sciences, Hyderabad, India; **Gilberto de Lima Lopes Jr**, Sylvester Comprehensive Cancer Center, Miami, FL; **Rebecca Alexandra Dent**, National Cancer Centre Singapore, Singapore; and **Shaheenah Dawood**, Mediclinic City Hospital, Dubai, United Arab Emirates.

Launched in 2009, the prestigious, highly selective, yearlong Leadership Development Program (LDP) provides midcareer oncologists with leadership training skills, advocacy experience, networking opportunities, and mentorship from senior American Society of Clinical Oncology (ASCO) members and leadership. In 2013, recognizing the growing number of international members and participants at ASCO’s annual scientific meeting and building on its efforts to develop oncology globally, ASCO welcomed its first international LDP participant. The program is currently open to 16 participants, only one of whom is chosen from outside the United States. LDP participants partake in person in three meetings held at ASCO headquarters and special sessions at the Annual Meeting.

LDP curriculum includes strategy development, conflict management, interpersonal effectiveness and teamwork, and media communication. Participants also meet ASCO administrative staff and learn how ASCO interacts with the US Food and Drug Administration, Capitol Hill, and advocacy organizations. LDP participants embark on ASCO strategic initiatives. Teams work on interactive learning projects, and their findings and formal recommendations are presented to the ASCO Board of Directors.

## UNIQUE CHALLENGES FOR LEADERS IN COUNTRIES OUTSIDE THE UNITED STATES

The distinct and diverse cultural and health care environments outside the United States present unique challenges for leadership. In developing countries with hierarchical health care systems, there is minimal focus on nurturing future leaders. There is no emphasis on leadership skills during training or professional practice. Most doctors focus on acquiring knowledge and technical skills rather than leadership qualities. Physicians mostly see themselves as healers. They tend to avoid conflicts or confrontation and are ill equipped to deal with corrupt and often hostile political environments. Rapid changes in the landscape of oncological care, ever-increasing cost of treatment, scarcity of resources, and little or no focus on prevention, add to the complexity of the problem. True to its pioneering role as a global organization, ASCO recognizes the strong need to develop and nurture international leaders in oncology. The inclusion of international participants in the LDP is indeed a big step toward building oncology leadership globally.

## PERSONAL EXPERIENCES

Traveling thousands of miles for a 2- to 3-day LDP training definitely requires time commitment. With the long hours of travel, we had to struggle to actively participate in the training sessions while enduring severe sleep deprivation due to jet lag. Certainly, time management, adaptability, and resiliency were the first few valuable lessons that we learned from LDP.

The program allowed us to better understand how a global society functions. One of the major challenges we had was to grasp the distinct health care education and delivery systems in the United States, because these are significantly different from those in our home countries. This was needed to effectively address the interactive learning projects entrusted to us by ASCO.

Our mentors and ASCO staff were extremely helpful and helped us navigate the challenges with ease. Leading meetings and assigning and completing tasks over long-distance calls were unique learning opportunities. Working and collaborating with diverse teams gave us an advantage of experiencing cultural norms. Creativity and new insights flourished in such environments, where we had different ideas and perspectives. Behaviors, work ethics, and communication styles were different variables that we learned to consider within cultural context. These helped us to be more flexible, open minded, and motivated. The interactive learning projects allowed an open exchange of creative and innovative ideas, many of which have been subsequently incorporated into ASCO offerings and policies.

LDP is enriching on many levels. It is an opportunity for US participants to get to understand and appreciate the other side of the world, offering a window to other cultures and perspectives. It was also truly gratifying to realize that our teams were highly interested in gaining insights into how ASCO can enhance education and quality of care internationally, thus making the experience mutually enriching.

## LEADERSHIP OPPORTUNITY EFFECTING REAL CHANGE IN INTERNATIONAL ONCOLOGY

LDP provided a strong foundation of leadership skills. It taught us how to build relationships, manage conflicts, set goals, and fulfill a vision. With the leadership opportunity, we became more effective, vocal, and confident in our involvement at the national and international levels.

LDP places graduates on ASCO committees or working groups. Some of us have served on the International Affairs Committee that focuses on improving global cancer care. The Committee supports the professional development, education, and research capacity of international members. We had the wonderful opportunity to participate in various international activities of ASCO, getting a glimpse of the immense potential of global oncology leadership.

Our active involvement and enhanced engagement with ASCO increased the awareness of our international colleagues in its global oncology programs. These allowed us to be ambassadors representing ASCO in our home countries and local communities. These gave us the valuable opportunity to be involved in various international courses, to participate in improving global medical oncology training, and to advance cancer care using ASCO’s various resources.

The program is strongly focused toward nurturing our potential and acquiring the skills required for effective leadership. These skill sets are applicable across diverse cultures and help us overcome many hurdles in our own unique environments. The program has enabled us to successfully undertake various leadership roles in our home countries and organizations and has also empowered us to effectively mentor our colleagues and juniors to take on various leadership roles. LDP taught us ways to approach challenging situations. It enhanced our critical thinking, decision making, and capability to manage complexities. These career skills prepare us to collaborate with government, other international organizations, and various stakeholders in global oncology.

## LONG-TERM AND FAR-REACHING BENEFITS

Quality leadership is a combination of the right qualities and the right training. It is key not only for any career but also in day-to-day life. LDP allowed us to identify our strengths and weaknesses and to know self-limitations that can help us perform more effectively in cross-cultural settings. Before one becomes an effective leader, one has to develop self-awareness. Living by our values, being self-disciplined, having a good work ethic, and being held fully accountable for our responsibilities are qualities that enable us to develop into better and effective global leaders. The long-lasting and continuing effect of the program continues to shape ASCO with its ever-increasing portfolio of international members and activities.

ASCO’s evolution as an international organization positively affects global cancer care. International participation in ASCO’s LDP should remain strong. It is essential that ASCO continue to support professional development and training of global oncology leaders (Table 1).

**Table 1:**
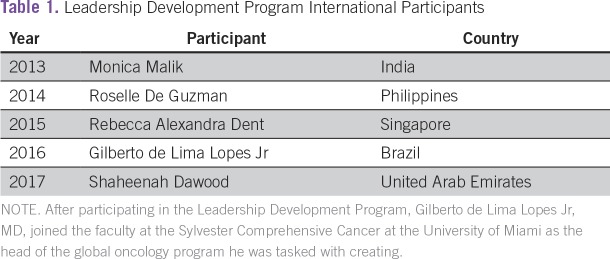
Leadership Development Program International Participants

